# The Antagonism of Corticotropin-Releasing Factor Receptor-1 in Brain Suppress Stress-Induced Propofol Self-Administration in Rats

**DOI:** 10.3389/fnbeh.2021.775209

**Published:** 2021-12-02

**Authors:** Zhanglei Dong, Gaolong Zhang, Saiqiong Xiang, Chenchen Jiang, Zhichuan Chen, Yan Li, Bingwu Huang, Wenhua Zhou, Qingquan Lian, Binbin Wu

**Affiliations:** ^1^Department of Anesthesiology, Perioperative and Pain Medicine, The Second Affiliated Hospital and Yuying Children’s Hospital of Wenzhou Medical University, Wenzhou, China; ^2^Clinical Research Unit, The Second Affiliated and Yuying Children’s Hospital of Wenzhou Medical University, Wenzhou, China; ^3^Medical School, Institution of Reproductive Medicine, Nantong University, Nantong, China; ^4^Department of Neurology, The Second Affiliated Hospital and Yuying Children’s Hospital of Wenzhou Medical University, Wenzhou, China; ^5^Zhejiang Provincial Key Lab of Addiction, Ningbo Kangning Hospital, School of Medicine, Ningbo Universtiy, Ningbo, China

**Keywords:** stress, CRF, D1 receptor, propofol, addiction

## Abstract

Propofol addiction has been detected in humans and rats, which may be facilitated by stress. Corticotropin-releasing factor acts through the corticotropin-releasing factor (CRF) receptor-1 (CRF1R) and CRF2 receptor-2 (CRF2R) and is a crucial candidate target for the interaction between stress and drug abuse, but its role on propofol addiction remains unknown. Tail clip stressful stimulation was performed in rats to test the stress on the establishment of the propofol self-administration behavioral model. Thereafter, the rats were pretreated before the testing session at the bilateral lateral ventricle with one of the doses of antalarmin (CRF1R antagonist, 100–500 ng/site), antisauvagine 30 (CRF2R antagonist, 100–500 ng/site), and RU486 (glucocorticoid receptor antagonist, 100–500 ng/site) or vehicle. The dopamine D1 receptor (D1R) in the nucleus accumbens (NAc) was detected to explore the underlying molecular mechanism. The sucrose self-administration establishment and maintenance, and locomotor activities were also examined to determine the specificity. We found that the establishment of propofol self-administration was promoted in the tail clip treated group (the stress group), which was inhibited by antalarmin at the dose of 100–500 ng/site but was not by antisauvagine 30 or RU486. Accordingly, the expression of D1R in the NAc was attenuated by antalarmin, dose-dependently. Moreover, pretreatments fail to change sucrose self-administration behavior or locomotor activities. This study supports the role of CRF1R in the brain in mediating the central reward processing through D1R in the NAc and provided a possibility that CRF1R antagonist may be a new therapeutic approach for the treatment of propofol addiction.

## Introduction

Propofol is an intravenous anesthetic mainly used for anesthesia induction and sedation in more than 50 countries. However, with the sedative and relaxing effect of propofol, its recreational abuse and dependence have risen (Earley and Finver, 2013). The abuse and misuse of propofol have recently become a social problem in many countries, and anesthesiologists are the main potential abusers, who usually suffered great pressure from daily clinical work (Wischmeyer et al., 2007; Fry et al., 2015; Park et al., 2015). We have demonstrated propofol as a substance for addiction in animals with the self-administration model, which was mediated by dopamine D1 receptor (D1R) in the nucleus accumbens (NAc) (Lian et al., 2013). We also found that propofol self-administration behavior was prompted by glucocorticoid—a stress hormone released from the hypothalamic-pituitary-adrenal (HPA) axis under the regulation of D1R in the NAc in rats (Wu et al., 2016, 2018). This effect can be attenuated by the intraperitoneal injection of RU486, an antagonist of the glucocorticoid receptor (GR) (Wu et al., 2016). However, whether the corticotropin-releasing factor (CRF) participates in the modulation of propofol self-administration behavior remains to be elucidated.

Previous studies demonstrated that stress increased the susceptibility of an individual to drug abuse (Sinha, 2008). The self-administration of psychomotor stimulants in animals were escalated after intermittent exposure to various stressors such as amphetamines and cocaine (Newman et al., 2018). The neuropeptide of CRF is a key modulator of physiological endocrine and behavior during stress, as well as the first identified central initiator of the classic HPA axis stress neuroendocrine response (Roberto et al., 2017). The CRF-containing system not only includes the HPA axis, but many findings also confirmed that stress-induced drug seeking can be mediated by extrahypothalamic CRF sites in the brain (Lasheras et al., 2015). As such, CRF has been a candidate target for the interaction between stress and drug abuse, playing a critical role in stress-escalated drug taking (Koob and Volkow, 2010; Newman et al., 2018).

Corticotropin-releasing factor signaling *via* CRF receptor-1 (CRF1R) and CRF receptor-2 (CRF2R), and is a preferential agonist for CRF1R over CRF2R. Corticotropin-releasing factor receptors widely signal throughout the brain, such as the ventral tegmental area (VTA), NAc, amygdala, and bed nucleus of the stria terminalis (Baumgartner et al., 2021). It was reported that the CRF-induced increase in the activity of dopamine (DA) neurons in the VTA might enhance release in the NAc, which potentiates drug-seeking behaviors and the response to reward (Wanat et al., 2008). To investigate the modulation of CRF in the central system for addiction, the ventricle injection of CRF was adopted in many studies. Both acute and chronic blockade of CRF1R by the lateral ventricle injection of CRF1R antagonist attenuated cocaine-induced DA release in the NAc (Lodge and Grace, 2005). Antagonizing CRF1R but not CRF2R blocked morphine-induced conditioned place preference (CPP) (Lasheras et al., 2015). These findings include the results of pharmacological and transgenic studies, indicating that CRF1R and CRF2R have differential roles in regulating addiction behavioral response (Valdez et al., 2004; Roberto et al., 2017). Corticotropin-releasing factor receptor-1 and CRF2R messenger RNA (mRNA) were detected in the VTA and NAc in rodents (Wischmeyer et al., 2007), in which both areas are pivotal in reward processing and drug abuse (Liu et al., 2020). Multiple studies suggested that drugs of abuse implement reward effects by increasing DA release in the NAc, where the dopaminergic afferent can be received from the VTA (Koob, 1999), and also, it was reported that CRF increases dopamine release in the NAc through CRF receptors (Lemos et al., 2012). Based on these findings, we assumed that CRF might regulate propofol self-administration behavior through the CRF receptors in the mesolimbic DA system.

In the present study, we adopted tail clip pretreatment to explore the effects of stress on propofol self-administration model establishment. After that, the role of CRF receptor and GR in the brain on propofol self-administration behaviors was examined with the tail clip-induced propofol self-administration model by the microinjection of antalarmin (a CRF1R antagonist), antisauvagine 30 (a CRF2R antagonist), and RU486 (an antagonist of GR) at the bilateral lateral ventricle. In addition, the pre-treatments on the expressions of D1R in the NAc, sucrose self-administration, and locomotor activities were also researched.

## Materials and Methods

### Animals

Adult male Sprague-Dawley rats weighing 300–350 g (14-week-old) were purchased from the Experimental Animal Center of Wenzhou Medical University. All procedures were consistent with the Guide for the Care and Use of Laboratory Animals and were approved by the Animal Care and Use Committee of Wenzhou Medical University. All operations were performed under anesthesia with sodium pentobarbital, and efforts were made to minimize the number of animals and suffering. The rats were housed in a temperature-controlled room individually under a 12-h light/dark cycle at 22–24°C, with free access to food and water. Only the rats that were successfully implanted with chronic indwelling catheters *via* the jugular vein and guide cannulae in the bilateral lateral ventricle were randomly assigned to continue the subsequent experiments.

### Drugs

Propofol in this study was obtained from Astra Zeneca (10 mg/ml, Diprivan, Italy), and was prepared daily for self-administration behavioral training. A single dose of 1.7 mg/kg/injection was used for the training as described in previous studies (McAulliffe et al., 2006). The CRF1R antagonist antalarmin (Axon Medchem, the Netherlands), CRF2R antagonist (Tocris Bioscience, Ellisville, MO, United States), and GR antagonist RU486 (Sigma-Aldrich, St-Louis, MO, United States) were dissolved in artificial cerebrospinal fluid (ACSF) (Zhongxing Chemical Reagent Co., Ltd., Zhejiang, China) (122.5 mM NaCl, 3.5 mM KCl, 25 mM NaHCO3, 1 mM NaH2PO4, 2.5 mM CaCl2, 1 mM MgCl2, 20 mM glucose, 1 mM ascorbic acid (pH: 7.40, 295-305 mOsm) (Yarur et al., 2020). The doses of the agents adopted in the present study were determined on previous behavioral studies (Blacktop et al., 2011; Taslimi et al., 2018).

### Surgeries

The implantations of intravenous catheters were performed as described previously (Zhou et al., 2007). The rats were implanted with the chronically indwelling intravenous catheters under sodium pentobarbital anesthesia (40 mg/kg) and the catheter were flushed daily with 0.2 ml saline-heparin solution to maintain the patency. Meanwhile, the rats were treated with penicillin B once a day through the implanted catheter to prevent infection during the recovery period for at least 7 days. The intra-lateral ventricle injections (A/P −0.8 mm, M/L ± 1.4 mm, D/V—3.5 mm) were done through bilaterally implanted guide cannulae (20 gauge, Small Parts Inc., United States) (Biagioni et al., 2006).

### Tail Clip Procedure

The acute pain induced by the tail clip test was according to a tail clip procedure described in a previously published study (Goebel-Stengel et al., 2014; Lee et al., 2017). The rats were put in a custom-made acrylic cylinder and given 10 min to accustom themselves to the new environment. An alligator clip exerting a force of 2.5 N was manually applied to the tail at a position approximately 2.5 cm proximal to the tail tip to induce pain for 2 min. The force was measured by attaching a flexible force sensor to the tail (FSR-400, Interlink Electronics, CA, United States). We observed that the tail clip pretreatment did not cause any apparent physical damage in the rats.

### Intra-Lateral Ventricle Microinjection Procedure

To evaluate the effects of the agents on the establishment and maintenance of tail clip-induced propofol self-administration behavior, sucrose self-administration, and locomotor activities, the rats were treated with ACSF (vehicle), antalarmin (100 and 500 ng/site), antisauvagine 30 (100 and 500 ng/site), or RU486 (100 and 500 ng/site) 10 min before the behavior test session. The microinjection in the lateral ventricle was delivered through the previous indwelling infusion cannula with a microinjection pump (MD-1001, Bioanalytical System Inc., West Lafayette, IN, United States) in a volume of 0.25 μL over 5 min.

### Self-Administration Apparatus

The apparatus for propofol self-administration (Ningbo Addiction Research and Treatment Center, Zhejiang, China) behavior training has been described in a previous study (Dong et al., 2021). Briefly, the apparatus was accompanied with custom-made operant boxes that sized 30 cm × 30 cm × 30 cm and equipped with two nose-poke operanda (active nose-poke and inactive nose-poke) located 5 cm above the floor with a yellow LED light inside each nose-poke hole. The rats were trained for the self-administration of propofol through the jugular injection with a 5-ml syringe that was attached to a special pump at the speed of 1.2 ml/min. The rats would receive a propofol infusion of 1.7 mg/kg after one active nose-poke as a reward (fixed ratio 1, FR1), which was paired with a 5-s extinguishing of the house light and the noise from the propofol infusion pump. No injection was given after an inactive nose-poke. Each active nose-poke was followed by a 30-s time-out period, no injection or reward would be given even if nose-poke occurred, both house light and the lights in the active and inactive nose-poke hole remained illuminated when active or inactive nose-poke occurred during the time-out period, and the numbers of nose-poke would be recorded. All the behavioral training sessions were automatically recorded by the computer.

### Propofol Self-Administration Training

The rats were trained under a fixed ratio 1 (FR1) schedule with a daily 3-h training session for 14 consecutive days, and the training session ended when the 3-h training time or 100 propofol infusions was reached. The numbers of active nose-poke and propofol infusion increased to a stable stage as the training proceeded till a successive 14-day training, and the inactive nose-poke decreased to a minimal level. The successful establishment of the propofol self-administration behavior model was determined by the variability of less than 10% in the last three sessions (Filip and Frankowska, 2007). The rats that did not reach the criteria were excluded in this step. There were 25 rats trained for establishing propofol self-administration behavior model with (the stress group, *n* = 12) or without tail-clip stimulation (the control group, *n* = 12), and one rat was ruled out. Another 58 rats received a 2 min tail-clip stressful stimulation 30 min before propofol self-administration training, and two rats were excluded. Finally, there were 56 rats randomly assigned to the groups that received a lateral ventricle injection of ACSF, antalarmin, antisauvagine 30, or RU486 (the vehicle group, *n* = 8; the antalarmin group *n* = 8; the antisauvagine 30 group, *n* = 8; the RU486 group, *n* = 8).

### Specific Experiments

***Experiment 1***: To explore the role of CRF1R in the brain on the stress-induced propofol self-administration behavior model, the rats that received tail clip-induced propofol self-administration training were microinjected at the bilateral lateral ventricle with ACSF (vehicle) or antalarmin (100 and 500 ng/site) 10 min prior to the behavior test session on day 15.

***Experiment 2***: To investigate the role of central CRF2R on stress-induced propofol self-administration behavior, the tail clip-induced propofol self-administration training rats were randomly assigned to the groups that received microinjection at the bilateral lateral ventricle with ACSF (vehicle) or antisauvagine 30 (100 and 500 ng/site), 10 min prior to the behavior test session on day 15.

***Experiment 3***: To evaluate the effects of GR on the tail clip-induced propofol self-administration behavior, the training rats received ACSF (vehicle) or RU486 (100 and 500 ng/site) pretreatment 10 min prior to the behavior test session on day 15.

### Sucrose Self-Administration Training

The rats were trained for sucrose self-administration daily for food reward under an FR1 schedule during a 0.5-h session consecutively for 7 days (*n* = 6). The paradigm for sucrose self-administration was similar to the paradigm of propofol, but the reward was changed to a 45-mg sucrose pellet (Dustless precision pellets, Bio-Serv, United States) that was delivered *via* a special cup after an active nose-poke, and inactive nose-pokes did not result in any programmed consequence. The sessions ended after either 0.5 h or if 100 pellets occurred, and the behavioral training sessions were automatically recorded by a computer. All rats reached the criteria of the successful establishment of the sucrose self-administration behavioral model. The rats were trained to establish a sucrose self-administration behavior model with (the stress group, *n* = 6) or without tail clip stimulation (the control group, *n* = 6) for 2 min to investigate the effects of the tail clip stressful stimulation on the establishment of sucrose self-administration behavioral model. The other 42 rats that received the 2-min tail clip stimulation 30 min before daily sucrose self-administration training were microinjected with ACSF (vehicle), antalarmin, antisauvagine 30, or RU486 at the bilateral lateral ventricle injection on day 8 to examine the maintenance of the sucrose self-administration behavioral model (the vehicle group, *n* = 6; the antalarmin group *n* = 6; the antisauvagine 30 group, *n* = 6; the RU486 group, *n* = 6).

### Locomotor Activity

The testing of the locomotor activity was performed in an experimental box with the size 30 cm × 40 cm × 50 cm, and was equipped with an image tracking and processing system. The rats received tail clip stressful pretreatment and a microinjection of ACSF (vehicle), antalarmin, antisauvagine 30, or RU486 at the bilateral lateral ventricle as described above prior to the locomotor activity testing, which was followed by a 1-h acclimation and a 3-h test session. The path length of the rats was monitored by a digital camera on the top of the experimental box and recorded automatically by the camera tracking system (the vehicle group, *n* = 6; the antalarmin group *n* = 6; the antisauvagine 30 group, *n* = 6; the RU486 group, *n* = 6).

### Western Blot Analysis

The NAc was removed immediately after the completion of the propofol self-administration test on day 15 (*n* = 4). The rats were deeply anesthetized with sodium pentobarbital (40 mg/kg) and then were euthanized by decapitation. The brain was removed and the NAc was dissected out (Paxinos and Waston, 2007). The total protein was extracted from the NAc and the protein concentration was measured with a bicinchoninic acid (BCA) protein assay kit (Beyotime, Shanghai, China). After being denatured at 100°C for 10 min, 40 μg protein was loaded on sodium dodecyl sulfate-polyacrylamide gel electrophoresis (SDS-PAGE) for electrophoretic separation, which was followed by the transfer to polyvinylidene fluoride (PVDF) membranes and non-specific binding site blocked with 5% skim milk (Merk) for 2 h at room temperature (RT). The band was incubated in primary D1 antibody (rabbit, 1:1,000, Abcam, Cambridge, MA, United States) at 4°C overnight, and in the secondary antibody (goat anti-rabbit, 1:5,000, Bioworld, Minnesota, United States) that was diluted in tris-buffered saline (TBST) for 2 h at RT. Glyceraldehyde-3-phosphate dehydrogenase (GAPDH) was adopted as the internal control. Finally, the band was visualized with an enhanced chemiluminescence (ECL) solution (GE Healthcare, Chicago, IL, United States) and photomicrographed with Image Quant LAS 4000 mini (GE Healthcare, Chicago, IL, United States).

### Statistical Analysis

The continuous data were presented as mean ± SD, and the normality of data distribution was tested. For the normally distributed data, one-way ANOVA was adopted for the analyses between multiple groups when the data also meets the homogeneity of variance, and Dunnett’s *post hoc* test was used for multiple comparisons. The data of the repeated measurements were analyzed with the two-way ANOVA of repeated measures. The Kruskal–Wallis test was used for data that were non-normally distributed. Statistical calculations were performed with SPSS 25.0 (SPSS Inc., Chicago, United States), and *p*-value < 0.05 was considered significant.

## Results

### Stress Stimulation Facilitated the Establishment of Propofol Self-Administration Behavior Under the FR1 Schedule

**FIGURE 1:**
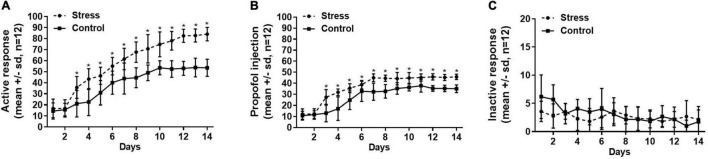
The numbers of active nose-poke responses **(A)**, propofol infusions **(B)**, and inactive nose-poke responses **(C)** were compared between the stress group (with tail clip pretreatment) and the control group (without tail clip pretreatment) by using the two-way ANOVA of repeated measures, showing that the active nose-poke responses and propofol infusions were higher in the stress group than the control group (active nose-poke response *p* < 0.001, infusion *p* < 0.001, *n* = 12), and the number of inactive nose-poke response was not significantly different in both groups (*p* = 0.16). SD, standard deviation, *p < 0.05.

Figure 1 shows the rats in both stress group that suffered tail clip stressful pretreatment and the control group that did not receive the tail clip stimulation successfully established propofol self-administration behavior within 14 days, presenting a significant increase in the active nose-poke response and propofol infusions, and a decrease in the inactive response. However, the numbers of active nose-poke responses and propofol infusions were higher in the stress group than the control group (Figure 1A, active nose-poke response, *F* = 8.975, *p* < 0.001; Figure 1B, infusion, *F* = 4.882, *p* < 0.001), but the number of inactive nose-poke responses was not significantly different between the two groups (Figure 1C, *F* = 1.875, *p* = 0.16) with significant differences. The results suggested that the establishment of propofol self-administration under the FR1 schedule was facilitated by the tail clip stressful pretreatment.

### Different Effects of the Bilateral Microinjection of Antalarmin, Antisauvagine 30, and RU486 at the Lateral Ventricle on Stress-Induced Propofol Self-Administration Behavior

**FIGURE 2:**
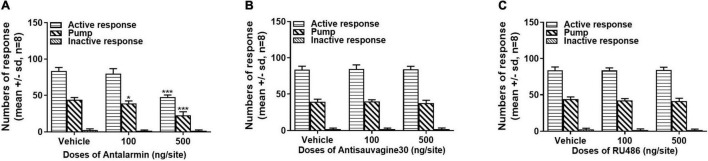
**(A)** Intra-lateral ventricle injection of corticotropin-releasing factor (CRF) receptor-1 (CRF1R) antagonist antalarmin attenuated active nose-poke responses and propofol infusions in a dose-dependent manner (active nose-poke response *p* < 0.001, infusion *p* < 0.001, *n* = 8) in the rats who received tail clip stimulation before daily training and the testing session, while the numbers of inactive nose-poke responses did not show a significance compared with the vehicle group (*p* = 0.32). **(B)** Intra-lateral ventricle pretreatment of the CRF receptor-2 (CRF2R) antagonist antisauvagine 30 did not alter the active nose-poke responses (*p* = 0.94), propofol infusions (*p* = 0.39), or inactive nose-poke response (*p* = 0.95). **(C)** The pretreatment with the glucocorticoid receptor (GR) antagonist RU486 at the lateral ventricle was unlikely to affect the active nose-poke responses (*p* = 0.95), propofol infusions (*p* = 0.26), or inactive nose-poke responses (*p* = 0.59). The normally distributed data were analyzed by one-way ANOVA with Dunnett’s *post hoc* test for multiple comparisons, otherwise were analyzed with a Kruskal–Wallis test. SD, standard deviation.*p < 0.05, ***p < 0.001.

The rats that were trained to have propofol self-administration behavior with the tail clip stressful pretreatment were either microinjected with ACSF (vehicle) or antalarmin (100 and 500 ng/site) at the bilateral lateral ventricle 10 min before the propofol self-administration behavior testing session on day 15. It was found that antalarmin dose-dependently attenuated the numbers of active nose-poke responses and propofol infusions compared with the vehicle group (Figure 2A, active nose-poke response, *H* = 15.965, *p* < 0.001; infusion, *F* = 65.653, *p* < 0.001), but the inactive nose-poke was not significantly affected (*F* = 1.195, *p* = 0.32). Whereas, no significant difference was found after the rats were pretreated with antisauvagine 30 (100 and 500 ng/site) in either active nose-poke (Figure 2B, *F* = 0.062, *p* = 0.94), propofol infusions (*F* = 0.997, *p* = 0.39) or inactive nose-poke (*F* = 0.057, *p* = 0.95) compared with the vehicle group. These results indicated that CRF1R but not CRF2R in the brain participated in the process of propofol self-administration modulation.

To further explore the role of central GR on tail clip-induced propofol self-administration behavior, the rats were bilaterally intra-lateral ventricle microinjected with the vehicle or RU486 (100 and 500 ng/site). All the pretreatments failed to alter the numbers of active nose-poke responses (Figure 2C, *F* = 0.051, *p* = 0.95), propofol infusions (*F* = 1.460, *p* = 0.26), or inactive nose-poke responses (*F* = 0.551, *p* = 0.59).

### The Expressions of D1R in the NAc Were Attenuated by Bilateral Lateral Ventricle Microinjection of Antalarmin, Not Antisauvagine 30 or RU486

**FIGURE 3:**
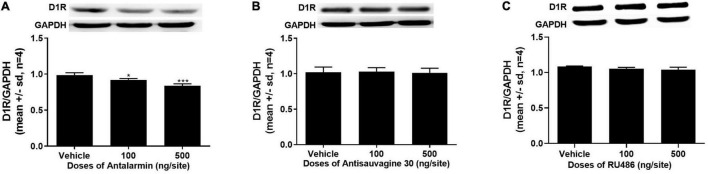
**(A)** The bilaterally intra-lateral ventricle injection of antalarmin significantly attenuated the expression of dopamine D1 receptor (D1R) in the nucleus accumbens (NAc) at the doses of 100 ng/site (*p* = 0.016) and 500 ng/site (*p* < 0.001) in the rats who received tail clip stimulation prior to daily training and the testing session (*n* = 4). **(B)** The expression of D1R in the NAc was not altered by the pretreatment of antisauvagine 30 at the bilateral lateral ventricle (*p* = 0.92). **(C)** The pretreatment with RU486 at the bilateral lateral ventricle did not significantly change the D1R expression in the NAc (*p* = 0.070). The data was analyzed with one-way ANOVA with Dunnett’s *post hoc* test for multiple comparisons. SD, standard deviation. *p < 0.05, ***p < 0.001.

The expressions of D1R in the NAc were detected after the completion of the tail clip pretreated propofol self-administration behavior testing session on day 15. The ANOVA analysis found that antalarmin significantly inhibited the expression of D1R in the NAc at the doses of both 100 and 500 ng/site (Figure 3A, *F* = 28.267, *p* < 0.001). However, there was no significant difference detected in the groups that were pretreated with antisauvagine 30 (Figure 3B, *F* = 0.087, *p* = 0.92) or RU486 compared with the vehicle group (Figure 3C, *F* = 3.631, *p* = 0.070).

### Stress Stimulation Failed to Affect the Establishment of the Sucrose Self-Administration Behavioral Model Under the FR1 Schedule

**FIGURE 4:**
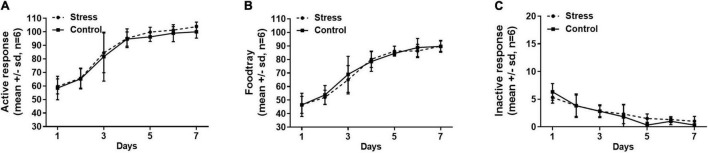
The numbers of active nose-poke responses **(A)**, food tray **(B)**, and inactive nose-poke responses **(C)** were compared between the stress group and the control group, indicating that neither the active nose-poke responses (*p* = 0.88), food tray (*p* = 0.76), nor the number of inactive nose-poke responses (*p* = 0.62) was affected by the tail clip stressful stimulation. The data were analyzed with repeated measures of ANOVA. SD, standard deviation.

The establishment of sucrose self-administration in the stress group and the control group was shown in Figure 4. The numbers of active nose-poke responses and sucrose pellets (food tray) increased as the training proceeded and stabilized at a high level in both the stress and control groups, and the inactive nose-poke responses decreased to a minimal level after the 7-day training. We found that neither active nose-poke response (Figure 4A, *F* = 0.109, *p* = 0.88), food tray (Figure 4B, *F* = 0.330, *p* = 0.76), nor inactive nose-poke response (Figure 4C, *F* = 0.743, *p* = 0.62) was changed in the stress group compared with the control group.

### Microinjection of Antalarmin, Antisauvagine 30, or RU486 at Lateral Ventricle Did Not Alter Sucrose Self-Administration Behavior or Locomotor Activities

**FIGURE 5:**
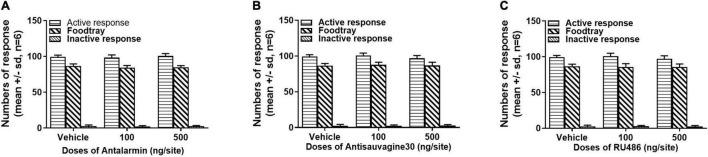
The intra-lateral ventricle injection of antalarmin **(A)**, antisauvagine 30 **(B)**, or RU486 **(C)** bilaterally were unlikely to affect the numbers of active nose-poke responses, food tray, and inactive nose-poke responses in the rats who received tail clip stressful stimulation. The normally distributed data were analyzed with one-way ANOVA, otherwise was analyzed with a Kruskal–Wallis test. SD, standard deviation.

**FIGURE 6:**
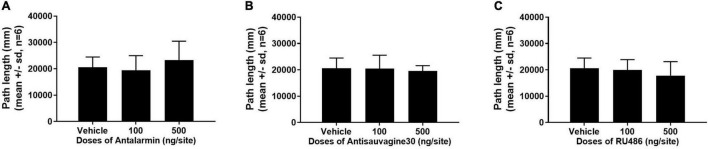
The pretreatment with antalarmin **(A)**, antisauvagine 30 **(B)**, or RU486 **(C)** at the bilateral lateral ventricle did not cause any difference that reached significance of the numbers of active nose-poke responses, food tray, and inactive nose-poke responses in the rats that receive tail clip stressful stimulation compared with the vehicle group. The normally distributed data were analyzed with one-way ANOVA, otherwise was analyzed with a Kruskal–Wallis test. SD, standard deviation.

The effects of the microinjections of antalarmin, antisauvagine 30, or RU486 at the bilateral lateral ventricle on sucrose self-administration and general locomotor activities were examined to further confirm the specificity of these pretreatments on propofol self-administration. The sucrose self-administration test was carried out on day 8. The results showed that all of the pretreatments failed to affect the numbers of active nose-poke responses (Figure 5, antalarmin, *F* = 0.669, *p* = 0.53; antisauvagine 30, *F* = 2.110, *p* = 0.16; RU486, *F* = 1.522, *p* = 0.25), food tray (antalarmin, *F* = 1.116, *p* = 0.35; antisauvagine 30, *F* = 0.166, *p* = 0.85; RU486, *F* = 0.077, *p* = 0.93), and inactive response (antalarmin, *F* = 0.227, *p* = 0.80; antisauvagine 30, *F* = 0.155, *p* = 0.86; RU486, *F* = 0.069, *p* = 0.93). Meanwhile, no pretreatments changed the general locomotor activities in the tail clip-stimulated rats as judged by the path length (Figure 6, antalarmin, *F* = 0.757, *p* = 0.49; antisauvagine 30, H = 114.047, *p* = 0.98; RU486, *F* = 0.651, *p* = 0.54).

## Discussion

In this study, it was found that the establishment of the propofol self-administration behavioral model was facilitated by tail clip stressful stimulation prior to the daily training session, which can be inhibited by the central administration of the antagonist of CRF1R antalarmin but was not affected by antisauvagine 30 or RU486 at the bilateral lateral ventricle. The detected expression of D1R in the NAc has been approved to be a crucial concern in mediating propofol self-administration behavior to explore potential molecular mechanisms (Lian et al., 2013). Our findings show that the expression of D1R in the NAc was notably attenuated by antalarmin but not by antisauvagine 30 or RU486. We also found that the establishment and maintenance of the sucrose self-administration behavioral model or general locomotor activities were not affected by all the pretreatments. Taken together, these findings support the role of CRF1R in the central nervous system in promoting propofol self-administration behavior, which may act through D1R in the NAc.

Addiction is conceptualized as a cycle of increasing the dysregulation of brain reward and anti-reward mechanisms that would result in a negative emotional state, subsequently contributing to compulsive drug-seeking behaviors (Koob, 2010). These counter-adaptive processes were hypothesized to be mediated by reward pathways and the brain stress systems. The neuropeptide CRF exerts a salient role in the neuronal networks for drug abuse initiation, escalation, and relapse (Newman et al., 2018). Previous research suggested that the chronic administration of drugs with dependence potential dysregulated the stress response mediated by CRF (Koob, 2010). The CRF was not only included in the HPA axis but also in the extrahypothalamic stress system in the brain. Also, the extrahypothalamic stress system includes the areas of VTA, NAc, and medial prefrontal cortex (mPFC) (Kelly and Fudge, 2018). The central administration of CRF at the ventricles reinstated heroin (Shaham et al., 1997), cocaine (Erb et al., 2006), and alcohol (Lê et al., 2002) seeking. Moreover, these reinstatements of drugs mimic the activation of behavioral responses to stress in rodents were blocked by competitive CRF receptor antagonists (Koob, 2010).

It is well known that the VTA and its dopaminergic projection to the NAc is one of the most important substrates for drug reward (Wischmeyer et al., 2007). Corticotropin-releasing factor was demonstrated to mediate the interaction between glutamatergic projection and dopaminergic neurons (Wise and Morales, 2010); induce glutamate release activates the mesocorticolimbic dopamine system (Wang et al., 2005); promote mesocorticolimbic DA release in the areas including the NAc; and cause lasting neural changes that may induce stress-escalated drug consumption (Park et al., 2015; Steger et al., 2020). Corticotropin-releasing factor plays roles through CRF1R and CRF2R but binds CRF1R with a 10-fold greater affinity compared with CRF2R (Hupalo et al., 2019). Previous studies implied that CRF1R but not CRF2R was involved in cocaine self-administration and morphine-induced CPP (Boyson et al., 2011; Lasheras et al., 2015). The NAc received dopaminergic projection from the VTA where CRF1R and CRF2R co-expressed on the dopaminergic neurons in rodents (Van Pett et al., 2000; Tan et al., 2017). The antagonism of CRF1R but not CFR2R in the VTA decreased footshock-induced reinstatement of cocaine seeking in rats and reduced the induction of locomotor cross-sensitization to cocaine (Blacktop et al., 2011; Boyson et al., 2014). As consistent with the above findings, the central administration of CRF1R antagonist antalarmin inhibited the tail clip-induced propofol self-administration but not the CRF2R antagonist antisauvagine 30, and activating CRF1R mimic the effect of footshock stress on reinstatement, and activation of the CRF2R did not (Blacktop et al., 2011). Both the acute and chronic blockade of CRF1R by the lateral ventricle injection with CRF1R antagonists attenuated cocaine-induced DA release in the NAc (Lodge and Grace, 2005). Despite the evidence above, the role of CRF1R and CRF2R on DA releasing remains controversial. Some studies indicated that CRF2R also was involved in the mediation of cocaine reinstatement, and the neuronal process of releasing DA and glutamate in the VTA (Wise and Morales, 2010). The footshock-induced reinstatement of cocaine-seeking was reported to be decreased by the VTA perfusion of CRF2R antagonists but not selective CRF1R antagonists (Mantsch et al., 2016), and cocaine induced a significant increase of VTA DA extracellular levels in the repeated stress rats at the presence of CRF1R antagonists (Sotomayor-Zárate et al., 2015). This discrepancy was ascribed to the distinct mechanisms underlying different abused drugs, and we speculate that the activation of CRF on the mesolimbic DA system might go through CRF1R but not CRF2R (Almela et al., 2012), all these needs to be further determined.

The mPFC is another vital site in the brain that contributes to drug addiction, innervating the VTA with glutamatergic efferent and receiving dopaminergic afferent (Tzschentke and Schmidt, 2003; Keramatian et al., 2019). Corticotropin-releasing factor receptor-1 is considered as the primary functional receptor subtype in the prefrontal cortex (PFC) (Perrin et al., 1995). We speculate that the mPFC may be inactivated after the central administration of the selective CRF1R antagonist antalarmin, and then causes the subsequent inhibition of dopaminergic neurons in the VTA, thereby resulting in the reduction in DA release in the NAc. In addition, CRF was reported to induce the rapid phosphorylation of the cyclic-AMP response element-binding protein (CREB) *via* the activation of CRF1R, while CRF2R played no discernable role (Stern et al., 2011). Along with the signaling pathway, NMDAR-D1R/ERK/CREB in the NAc was indicated to regulate reward-seeking behaviors (Kirschmann et al., 2014), and our previous findings stated that propofol self-administration behavior was regulated by ERK1/2 in the NAc (Wang et al., 2016). Therefore, we presume that the mediation of CRF1R on the D1R/ERK1/2/CREB signal pathway may be underlying the molecular mechanisms. This postulation is supported by our results in this study that the expression of D1R in the NAc was significantly inhibited by CRF1R antagonist antalarmin, but the effects on the expression on ERK1/2 and CREB needs to be examined in the following study. Although the tail clip-induced propofol self-administration behavior and expression of D1R in the NAc were not affected by the central pretreatment of RU486 in this study, our published study reported that both were attenuated by the systemic administration of RU486 (Wu et al., 2016). We believe that the difference in the approaches of agent delivery, doses, and the methods in establishing the propofol self-administration model might lead to the distinction on D1R expression in the NAc and propofol self-administration behavior.

Corticotropin-releasing factor receptors also take part in food addiction. The pretreatment with antalarmin reduced the stress-induced reinstatement of palatable food-seeking (Ghitza et al., 2006), but some other studies reported that the antagonism of CRF1R with R121919 or CP-154526 did not affect the response to food (Goeders and Guerin, 2000; Roberto et al., 2017). And we also found that sucrose self-administration was not affected by either tail clip stressful stimulation or the pretreatments of antalarmin, antisauvagine 30, or RU486. This seems to be contradictory between the previous findings and our study, which might be ascribed to the different food addiction testing models and the distinction of the mechanisms underlying the stage of self-administration and reinstatement in food addiction.

The limitations of this study should be mentioned. As previous studies indicated, the DA release in the NAc and VTA is regulated by CRF and may be a potential for CRF receptor mediating propofol self-administration behavior, we only detected the D1R expression in the NAc, but the changes of dopamine concentration and the downstream signal pathway of D1R in the NAc were not examined. Beyond that, the neuroadaptation in the VTA that is modulated by the glutamatergic afferent from the mPFC, the interactions between the presynaptic glutamate afferent, and the CRF receptor on postsynaptic on dopaminergic neurons in the VTA is also unclear. All these questions will be elucidated in the future.

## Conclusion

In conclusion, this study provides clear evidence that propofol self-administration behavior was facilitated by stressful stimulation, which could be inhibited by the central antagonism of CRF1R, not CRF2R or GR, and the neuronal process is mediated by the DA D1R in the NAc. This study emphasizes the role of CRF1R in the central reward processing and moreover, indicated that the CRF1R antagonist may provide a new therapeutic approach for the treatment of propofol addiction.

## Data Availability Statement

The raw data supporting the conclusions of this article will be made available by the authors, without undue reservation.

## Ethics Statement

The animal study was reviewed and approved by the Animal Care and Use Committee of Wenzhou Medical University.

## Author Contributions

BW, QL, and WZ were responsible for the study concept, design and assisted the interpretation of findings. ZD, GZ, SX, ZC, and BH contributed to the acquisition of animal data. BW and CJ performed the data analysis. BW, ZD, CJ, and YL drafted the manuscript. All authors critically reviewed content and approved version for final publication.

## Conflict of Interest

The authors declare that the research was conducted in the absence of any commercial or financial relationships that could be construed as a potential conflict of interest.

## Publisher’s Note

All claims expressed in this article are solely those of the authors and do not necessarily represent those of their affiliated organizations, or those of the publisher, the editors and the reviewers. Any product that may be evaluated in this article, or claim that may be made by its manufacturer, is not guaranteed or endorsed by the publisher.

## Abbreviations

CRF: corticotropin-releasing factor
CRF1R: CRF1 receptor
CRF2R: CRF2 receptor
GR: glucocorticoid receptor
D1R: dopamine D1 receptor
D2R: dopamine D2 receptor
FR1: fixed ratio 1
NAc: nucleus accumbens
VTA: ventral tegmental area
mPFC: medial prefrontal cortex
CPP: conditioned place preference.
